# Dynamic Covalent Chemistry Enabled Closed-Loop Recycling of Thermally Modified Polymer Membrane

**DOI:** 10.1021/acsapm.5c00491

**Published:** 2025-06-17

**Authors:** Ching Yoong Loh, Tianting Pang, Dengsong Zhang, Andrew D. Burrows, Ming Xie

**Affiliations:** † Department of Chemical Engineering, 1555 University of Bath, Bath BA2 7AY, United Kingdom; ‡ Department of Chemistry, College of Sciences, 34747 Shanghai University, Shanghai 200444, PR China; § Department of Chemistry, 1555 University of Bath, Bath BA2 7AY, United Kingdom

**Keywords:** dynamic covalent chemistry, Diels−Alder chemistry, circular economy, oil/water separation, electrospinning, sustainable membrane

## Abstract

The increasing demand for sustainable solutions to oil–water separation and end-of-life membrane disposal has prompted the development of recyclable membrane technologies. In this study, we present an innovative approach to fabricating closed-loop, recyclable nanofibrous membranes (RFMs) utilizing reversible covalent networks based on the Diels–Alder reaction. A methacrylate-based copolymer was synthesized via free radical polymerization, combining hydrophobic monomers for enhanced separation performance, with furan-functionalized monomers for recyclability. This copolymer was electrospun into a porous substrate and cross-linked with bismaleimide cross-linkers to form a dynamic covalent network. By incorporating postthermal modification to the nanofibrous membrane, the hydrophobicity and the membrane porosity can be optimized. The resulting RFM exhibited outstanding oil–water separation capabilities, achieving a pure oil flux of up to 1,187 LMH with a separation efficiency up to 99% in water–oil emulsions, as demonstrated in tests with dichloromethane and other oils. Notably, the RFMs maintained structural and chemical stability after two recycling cycles, with recycled membranes retaining fluxes of 474–1,187 LMH and efficiencies of 98.8–99.5%. Thermal and mechanical characterizations confirmed the great stability of the membranes, with the Diels–Alder reaction enabling depolymerization and reformation of the network without causing significant degradation. Additionally, the RFMs were recycled the third time, maintaining the fluxes (752 to 823 LMH) from the previous generation with a slight decrease in separation efficiency in dichloromethane-water emulsion separation (98.3 to 97%). By integrating dynamic covalent chemistry with scalable fabrication methods, RFMs represent a transformative step toward a circular economy in oil–water separation and broader wastewater treatment and resource recovery.

## Graphical abstract

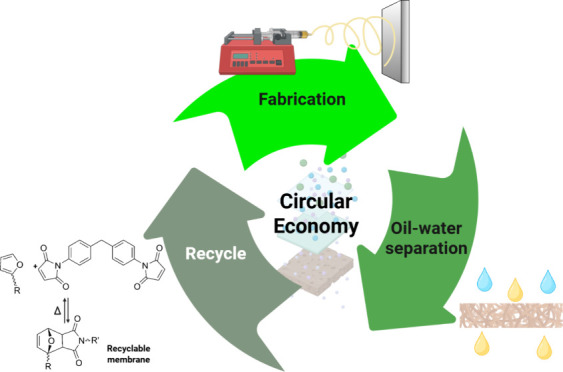

## Introduction

Oil pollution is a pervasive global issue driven by the rapid expansion of oil-related industries such as mining, petrochemical, and textile sectors, which frequently result in oil spills and the generation of substantial volumes of oily wastewater.^1–3^ For instance, a typical mining operation can produce up to 140,000 L of oily wastewater daily.^4^ Industrially discharged oily wastewater is among the most persistent pollutants, posing significant threats to aquatic ecosystems, human health, and contributing to irreversible climate change on a planetary scale.^5–7^ This underscores the urgent need to develop effective and efficient technologies for the separation of oil–water mixtures. There are several common techniques for addressing oil spills and separating oil–water mixtures, such as membrane separation,^8–10^ biological treatment,^11^ adsorption,^11^ air flotation,^12,13^ and gravity separation.^14^ Among these, membrane-based separation stands out due to its high separation efficiency, low energy consumption, and operational simplicity.^15^

Hydrophobic membranes are particularly promising for oily wastewater treatment, as their intrinsic properties enable effective water–oil separation. Various materials can be used to fabricate hydrophobic membranes, with polymeric materials dominating due to their high flux, low cost, and ease of manufacture.^16^ Commercial polymeric membranes, typically made from polysulfone (PSf), poly­(vinylidene fluoride) (PVDF), poly­(tetrafluoroethylene) (PTFE), and polyacrylonitrile (PAN), are widely used.^17–19^ However, these fossil-fuel-derived membranes inevitably reach their end-of-life due to fouling, mechanical wear, and defects, leading to significant membrane waste generation. For example, Landaburu-Aguirre’s research group estimated that over 840,000 spiral-wound membranes, equivalent to 14,000 tons, are disposed of annually from desalination plants alone.^20^

Disposal of end-of-life membranes is often categorized as general waste and typically involves landfill or incineration, both of which have significant environmental drawbacks.^20–22^ Landfill disposal can take years for membrane degradation, resulting in reduced land availability and potential leachate issues. Similarly, incineration generates greenhouse gases and toxic byproducts, including carcinogens, that adversely affect both human health and the environment.^23^ These challenges underscore the urgency of transitioning to more sustainable and environmentally friendly technologies.

A circular economy offers a promising solution by emphasizing waste minimization and cradle-to-cradle recycling, transforming waste into resources and closing the production loop. With increasing regulatory emphasis on sustainability, industries are encouraged to adopt circular practices that promote material reuse and reduce reliance on raw material consumption.^24,25^ As a result, modern manufacturing is progressively shifting toward sustainable material sourcing and waste reduction, aligning with global sustainability goals.^26,27^ By integration of recyclable materials into membrane technology, both challenges of managing oily wastewater and mitigating resource waste can be effectively addressed.

Dynamic covalent networks (DCNs) and covalent adaptable networks (CANs) present an innovative solution for closed-loop membrane recycling. These cross-linked polymers utilize dynamic covalent bonds that enable processability akin to thermoplastics while retaining the excellent properties of thermosets.^28–30^ These dynamic bonds can break and reform in response to external stimuli such as heat,^30,31^ light,^32^ pH changes,^33^ or the presence of specific catalysts,^34^ imparting properties like self-healing, reshaping, recycling, and stress relaxation. Due to their unique reversible-bond characteristics, DCNs can be an advanced solution to fabricate a recyclable membrane with high functional performance and great sustainability.^35–39^

We present an innovative application of DCNs to fabricate recyclable nanofibrous membranes (RFMs) using dynamic covalent chemistry via the Diels–Alder reaction. The innovative nature lies in the synthesis of a copolymer consisting of butyl methacrylate (BMA) and furfuryl methacrylate (FMA) as the monomer building block, where it combines hydrophobic BMA for efficient oil–water separation with furan-functionalized FMA for recyclability and forms the copolymer poly­(FMA-*co*-BMA) (PFB). While previous studies have explored dynamic covalent networks for recyclable membranes,^30,35,36^ this work introduces postheat treatment via hot-pressing as a facile and distinct step to enhance oil–water separation performance. The resulting membrane not only demonstrates a high separation efficiency but also offers closed-loop recyclability, overcoming the disposal challenges of conventional polymeric membranes. By utilizing electrospinning, a highly porous structure is achieved, combining satisfactory mechanical integrity with thermal stability compared to other studies.^40,41^ This work bridges the gap between sustainability and high-performance separation technologies, providing a transformative solution for addressing grand challenges in wastewater treatment and end-of-life membrane disposal management.

## Materials and Methods

### Materials

Furfuryl methacrylate (FMA, 97%), butyl methacrylate (BMA, 99%), 2,2′-azobis­(2-methylpropionitrile) (AIBN, 98%), 1,1′-(Methylenedi-4,1-phenylene)­bismaleimide (BMI, 95%), hexane (analytical grade), petroleum ether (ACS reagent), dichloromethane (DCM, ACS reagent), and SPAN-80 were purchased from Merck. Methanol (analytical grade) was purchased from VWR Chemicals. N,N-dimethylformamide (DMF, 99.8%, anhydrous) and toluene (99.85%, anhydrous) were purchased from Thermo Scientific Chemicals, UK. Deionized water was used in the preparation of aqueous solutions.

### Synthesis of Poly­(FMA-*co*-BMA)

Poly­(FMA-*co*-BMA) (PFB) was synthesized via free radical polymerization based on a slight modification from a previous study.^30^ FMA (15 mmol, 2.49 g), BMA (85 mmol, 12.09 g), and AIBN (1 mmol) were added into a twin-neck round-bottom flask with 50 mL of toluene under a dry argon gas environment. After purging the mixture with argon for 30 min at room temperature to eliminate air in the solution, the polymerization is initiated by heating the mixture to 80 °C for 24 h. Upon cooling after the reaction, PFB was precipitated and purified in methanol three times and dried in a vacuum oven at 80 °C overnight.

### Fabrication of RFM

In a typical fabrication process, recyclable nanofibre membranes (RFMs) were synthesized using an electrospinning apparatus composed of a syringe, syringe pump, high-voltage DC power supply, and stainless-steel collector. Initially, 1 g of PFB was dissolved in DMF to achieve a concentration of 30 wt %. Next, BMI was introduced into the mixture at a molar ratio of 0.5:1 (maleimide to furan functional groups). The resulting dope solution was heated to 140 °C and stirred vigorously for 15 min, followed by rapid cooling in a room-temperature water bath. Subsequently, the solution was reheated to 60 °C to induce cross-linking, which continued for 90 min. Following the cross-linking reaction, the solution was aspirated into a syringe connected to the high-voltage power supply. During the electrospinning process, the applied voltage was maintained at 16 kV, the needle-to-collector distance was set to 20 cm, and the solution flow rate was kept at 0.8 mL h^–1^. The collected fibers were then dried in an oven at 60 °C overnight to remove residual solvent and stabilize the final product.

To improve the separation performance of the RFM membrane, the dried membrane samples were then subjected to postheat treatment using a manual t-shirt press at hand-tight pressure with different temperatures and durations.

### Membrane Characterization

The synthesized copolymer and electrospun membranes were characterized using various analytical techniques. Specifically, molecular structure was analyzed by proton nuclear magnetic resonance (^1^H NMR) spectroscopy and Fourier-transform infrared (FT-IR) spectroscopy, including in situ FT-IR across a temperature range. Gel permeation chromatography (GPC) determined polymer molecular weight, while scanning electron microscopy (SEM) evaluated the morphology of the fiber and membrane. A gel content test was performed to evaluate the cross-link degree of the membranes. Thermal properties were evaluated using differential scanning calorimetry (DSC) and thermogravimetric analysis (TGA), and mechanical properties were assessed by dynamic mechanical analysis (DMA). Surface hydrophobicity was measured through water contact angle analysis.

Proton nuclear magnetic resonance (^1^H NMR) analysis of samples dissolved in deuterated chloroform with a 400 MHz Bruker NMR Spectrometer was performed.

Fourier-transform infrared (FT-IR) spectroscopy was performed using a Bruker FT-IR Spectrometer INVENIO to record the infrared spectra of the samples to determine the succession of the synthesis and fabrication. The spectral resolution was 4 cm^–1^, and 16 scans were recorded for the spectrum ranging from 4000 to 400 cm^–1^ for each sample after doing the background analysis.

X-ray photoelectron spectroscopy (XPS) was measured at 5 random points on the surface of the samples (Shimadzu AXIS Kratos Supra^+^, Kyoto, Japan). The relevant core levels of C 1s, O 1s, and N 1s were recorded with survey scans and high-resolution scans, respectively.

Gel permeation chromatography (GPC) was performed to determine the average molecular weight of the synthesized copolymer using an Agilent 1260 instrument. The samples were dissolved in tetrahydrofuran (THF), with a polystyrene calibration standard of polystyrene.

In situ FT-IR spectra were measured across different temperatures in a bespoke chamber. The temperature increase rate is 10 °C min^–1^, and 16 scans of the full spectra were recorded at each 10 °C interval until 180 °C with a spectral resolution of 4 cm^–1^.

Scanning electron microscopy (SEM) was performed to study the morphology of the electrospun membrane. Membrane samples were degassed in a negatively pressurized chamber overnight. The samples were coated with a layer of gold before carrying out SEM (Hitachi SU3900, Kyoto, Japan) to minimize charge buildup in the sample. ImageJ software is used to calculate the fiber diameter and size distribution of the fibrous membranes via the resulting SEM images.^42^

The gel content test was conducted using the following equation:

$$\mathrm{Gel}\;\mathrm{content}\%=100\times \frac{M_{1}}{M_{0}}$$ 1

where *M*_1_ and *M*_0_ are the weight of the dried sample after solvent immersion and the weight of the pristine sample.

Differential scanning calorimetry (DSC, Q20, TA Ltd., USA) was utilized to study the glass transition temperature of the polymer and the reversible Diels–Alder reaction of the fibrous membranes under a nitrogen environment. The samples ran through the first cycle from −50 to 150 °C at a heating rate of 10 °C min^–1^ and kept constant at 150 °C for 2 min. Next, the sample was cooled to −50 °C and heated to 170 °C with the same heating rate.

Dynamic mechanical analysis (DMA, TA System DMA 1, Mettler Toledo, USA) was used to perform the temperature sweep test and stress–strain test. The sample dimensions were kept consistent at around 10 × 8 × 0.11 mm. In the temperature sweep test, a heating rate of 2.5 °C min^–1^ was applied from 30 to 120 °C at a frequency of 1 Hz, with the storage modulus, loss modulus, and tan­(δ) recorded. Moreover, the stress–strain tests were determined with an elevated force of 1 N min^–1^ and the same sample dimensions.

Thermogravimetry analysis (TGA) was conducted on a Setaram Setsys Evolution TGA 16/18. The polymers and membrane samples were heated from room temperature to 600 °C with a heating rate of 10 °C min^–1^ in an atmospheric environment.

Water contact angle measurements were determined using DataPhysics OCA 25 with SCA 20 module base software. In a typical test, a 5 μL water drop was deposited on the surface of the membrane samples with a syringe needle. The water contact angle was recorded by using a high-resolution camera.

### Membrane Performance Evaluation

The membrane separation performance was carried out in a laboratory-based filtration apparatus, with an effective filtration area of 13.53 cm^2^. Each membrane samples were tested without any external pressure or suction. Different feed solutions, namely, pure oil (hexane, petroleum ether, and DCM) and their water emulsions, were used to pass through the membrane at room temperature (20 ± 1 °C), with the permeate flux, *J* (LMH), and the rejection rate, *R* (%) calculated as below:

$$J=\frac{V}{At}$$ 2

where *V*, *t*, and *A* are the volume of permeate (L), duration of the filtration period (h), and the effective surface area (m^2^).

$$R=\left(1-\frac{C_{P}}{C_{F}}\right)\times 100\%$$ 3

where *C*_*P*_ and *C*_*F*_ are the water contents of the permeate and feed (ppm), respectively.

The water-in-oil emulsion was prepared using 99 g of oil, 1 g of deionized water, and 0.25 g of surfactant (SPAN-80), where the solution was mixed vigorously overnight. The water content in the oil emulsion was measured using a Karl Fisher Titrator (Volumetric KF Titrator, V10S, Mettler Toledo, USA). The particle size of the water droplets in the water–oil emulsions was determined by dynamic light scattering (DLS) (Zetasizer Ultra, ZSU3305, Malvern, UK). The samples were measured five times and averaged.

### Closed-Loop Membrane Recycling

To demonstrate the closed-loop recycling capability of fabricated membranes, 1 g of contaminated RFMs was weighed and placed in a vial with DMF as the dissolving solvent. The concentration of the dope was adjusted to 28 wt % (26 wt % for the third recycling time), which was slightly lower than the original weight concentration. To mimic realistic conditions for used membranes, vegetable oil and sand were added to the membrane surface before they were put in the vials. The dope solution was heated at 140 °C for 20 min to promote depolymerization and later rapidly cooled in a room-temperature water bath. After filtering out the sands and decanting the adsorbed oil, the procedures for fabricating the membrane were then repeated to obtain the recycled nanofibrous RFM. The recycled RFM is denoted as RFM_Rx, with x indicating the number of recycles.

## Results and Discussion

### Dynamic Covalent Chemistry Enables Copolymer Synthesis

To fabricate the recyclable membranes, the copolymer PFB was synthesized via a free radical polymerization of the monomers BMA and FMA (Figure 1a). BMA was selected for its long alkyl chains, which impart increased flexibility and enhanced hydrophobicity, crucial for efficient oil–water separation. Conversely, FMA was chosen for its furan ring structure, which provides the conjugated diene required for the Diels–Alder reaction during the recycling process.

**Figure 1:**
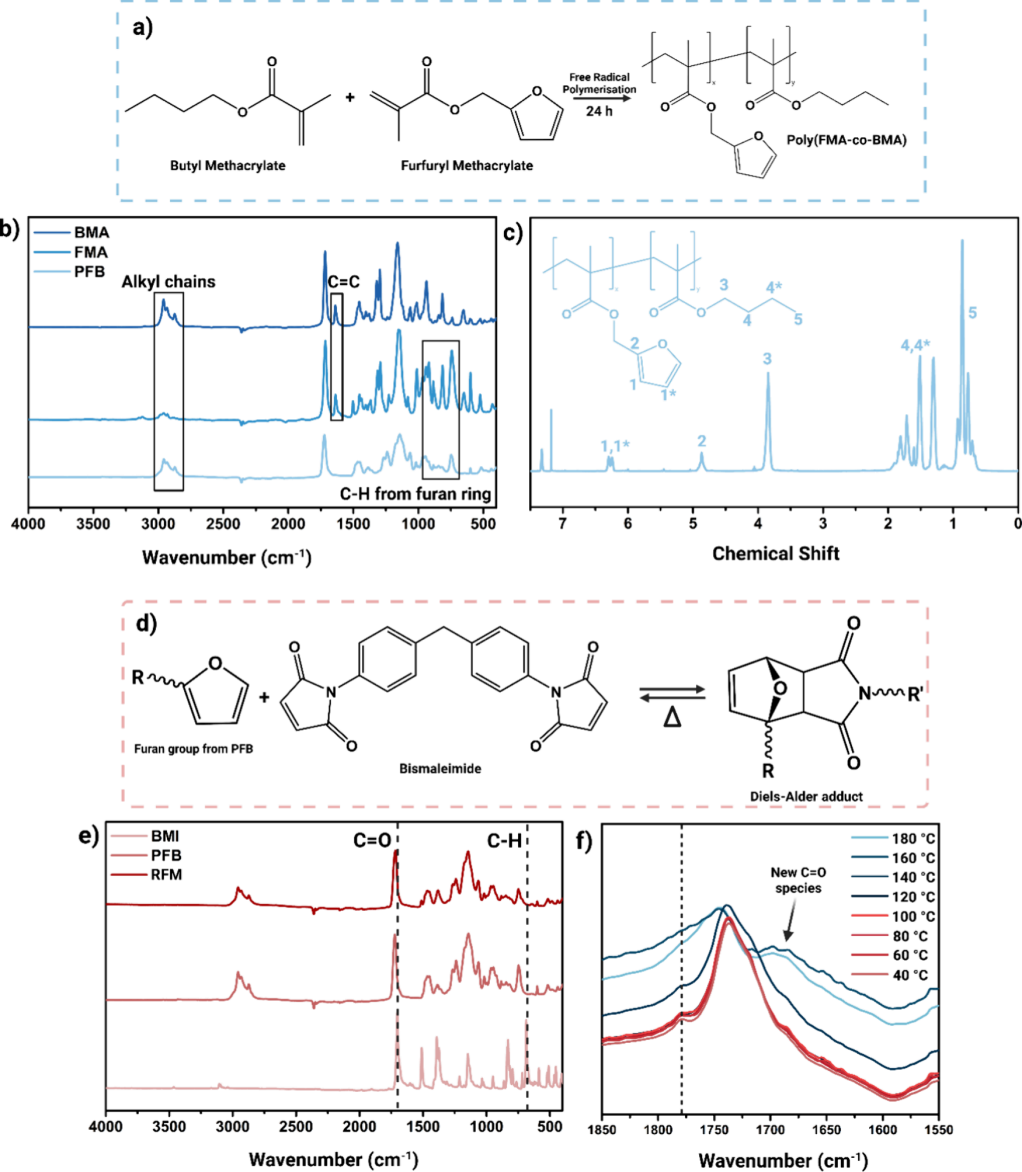
(a) Chemical reaction scheme, (b) FT-IR spectra, and (c) ^1^H NMR spectra of the copolymer PFB. (d) Proposed chemical reaction scheme for the Diels–Alder reaction of PFB and BMI, (e) the overall FT-IR spectra, and (f) in situ FT-IR spectra across the temperature of RFM.

The successful synthesis of PFB was confirmed by FT-IR spectroscopy (Figure 1b). A decrease in the peak at around 1650 cm^–1^, corresponding to the C=C stretching vibration of the unreacted methacrylate monomers, indicated the formation of the copolymer. Furthermore, the peaks in the range of 900–675 cm^–1^ were evident in both the FMA and PFB spectra. The peaks were attributed to the out-of-plane bending vibrations of C–H bonds in the furan ring, confirming the successful grafting of the furfuryl moiety onto the copolymer. The variations in peak intensities around 2950–2850 cm^–1^, with stronger signals observed for BMA and PFB compared to FMA, demonstrated the successful incorporation of alkyl chains from the butyl groups into the copolymer structure. Further confirmation of PFB formation was provided by ^1^H NMR spectroscopy (Figure 1c). Peaks corresponding to OCH_2_ protons from the furfuryl group (δ: 6.2 ppm) and aromatic protons of the furan ring (δ: 4.9 ppm) confirmed the presence of furfuryl groups derived from FMA within the copolymer. Similarly, signals for the OCH_2_ protons from the butyl group (δ = 3.8 ppm) were also detected. The resulting polymer sample has a butyl group to furfuryl group ratio of 1:5.87 (Figure S1). The molecular weight of PFB was 68 kDa (*M*_*w*_) and 28 kDa (*M*_*n*_) as determined by GPC, which gives a polydispersity index of 2.43.

PFB was further cross-linked with BMI, which served as a substituted diene source for the Diels–Alder reaction (Figure 1d). The copolymer and cross-linker were dissolved in DMF to form a dope solution, which was then used for electrospinning. The resulting nanofibrous membrane was designated as RFM_R0. FT-IR spectroscopy confirmed the successful cross-linking of BMI to the furan groups of PFB, as indicated by changes in two specific wavelength ranges (Figure 1e). The characteristic carbonyl (C=O) stretching vibration of the maleimide group in BMI was initially observed around 1730 cm^–1^, and the C–H out-of-plane bending at approximately 680 cm^–1^, diminished in the RFM_R0 spectra. This reduction in peak intensity verified the successful formation of cross-links between the maleimide and furan groups. Moreover, FT-IR at elevated temperatures was also conducted to confirm the retro DA reaction (Figure 1f). The first indication of retro DA was shown in a sudden disappearance of the peak around 1780 cm^–1^, which corresponds to the breaking of imide carbonyl linkages.^43^ On the other hand, the success of retro DA can also be confirmed with the potential oxidation of the maleimide group at higher temperatures. The emergence of a shoulder at around 1690–1650 cm^–1^ after 140 °C indicates the potential formation of new C=O carbonyl species or additional reactions occurring after retro DA. Gel content tests were performed to test the cross-linking degree of RFM_R0 in different organic solvents (DMF, toluene, DCM, and ethanol). The gel content was >96% in all the organic solvents, which shows the good membrane stability of RFM_R0 (Figure S2).

### RFM Exhibits Desirable Mechanical Integrity and Thermal Characteristic

The as-fabricated RFM typically exhibits an off-white color with a flexible nature (Figure 2a). To enhance the separation efficiency of the electrospun fibrous membrane, a hot-pressing technique was applied. This process significantly reduced the membrane porosity (Figure 2b–d), which is inversely correlated to separation performance. The untreated RFM exhibits a highly porous, interconnected fibrous network, while the hot-pressed RFM shows a denser structure with more tightly packed fibers, favoring the emulsion separation process.

**Figure 2:**
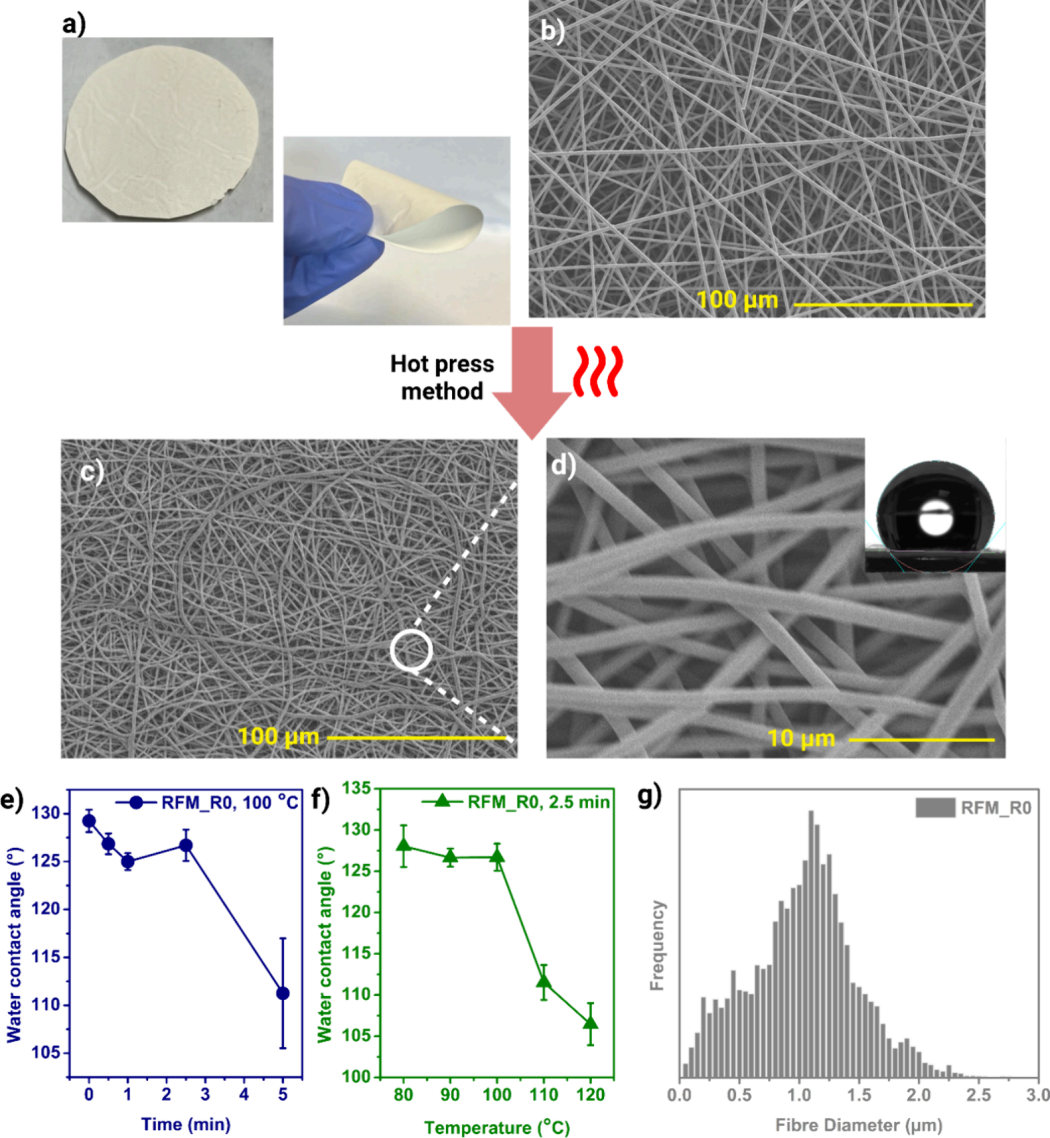
(a) Captured image of the RFM. SEM images of RFM_R0 (b) before and (c,d) after heat treatment (80 °C, 1 min). The relationship of the RFM_R0 heat treatment between water contact angle vs (e) time and (f) temperature. (g) Histogram of the fiber diameter distribution of RFM_R0.

Optimisation of the hot-pressing process was performed by varying the temperature and duration of treatment. Water contact angle measurements reveal the effects of hot pressing on surface hydrophobicity (Figure 2e). Initially, the RFM shows a high water contact angle (WCA), indicating a hydrophobic surface. However, prolonged exposure to elevated temperatures or increased pressing temperatures gradually reduced the water contact angle, suggesting a slight decline in hydrophobicity (Figure 2f). These findings indicate that the observed changes in porosity and WCA are primarily attributable to fiber rearrangement and densification, which aligns with previous studies on hot-pressed electrospun membranes.^44^ After the optimization, the remaining membrane samples will be fabricated by post-treating the electrospun RFMs via the hot-press method for 2.5 min at 80 °C. Consequently, the average fiber diameter of RFM_R0 was 1.05 ± 0.42 μm, as shown in Figure 2g, and the water contact angle was approximately 128.4° ± 2.3°. The structural change of RFM_R0 during 80 °C arises primarily from the physical rearrangement of polymer chains and fibers rather than the adjustment of cross-link density. As the operating temperature remained below the glass transition temperature (*T*_*g*_, 90 °C ∼ 100 °C) and retro-DA onset temperature (92.6 °C) (Figure 3b), the DA cross-links remain stable, while the heat and pressure from the t-shirt press induce fiber compaction and partial fusion, hence the decreased porosity. While 80 °C was selected as the minimum effective temperature for hot-pressing based on porosity and separation performance requirements, exploring lower temperatures (e.g., 60–70 °C) could further tune hydrophobicity and flux, particularly for both pure oil and emulsion systems.

**Figure 3:**
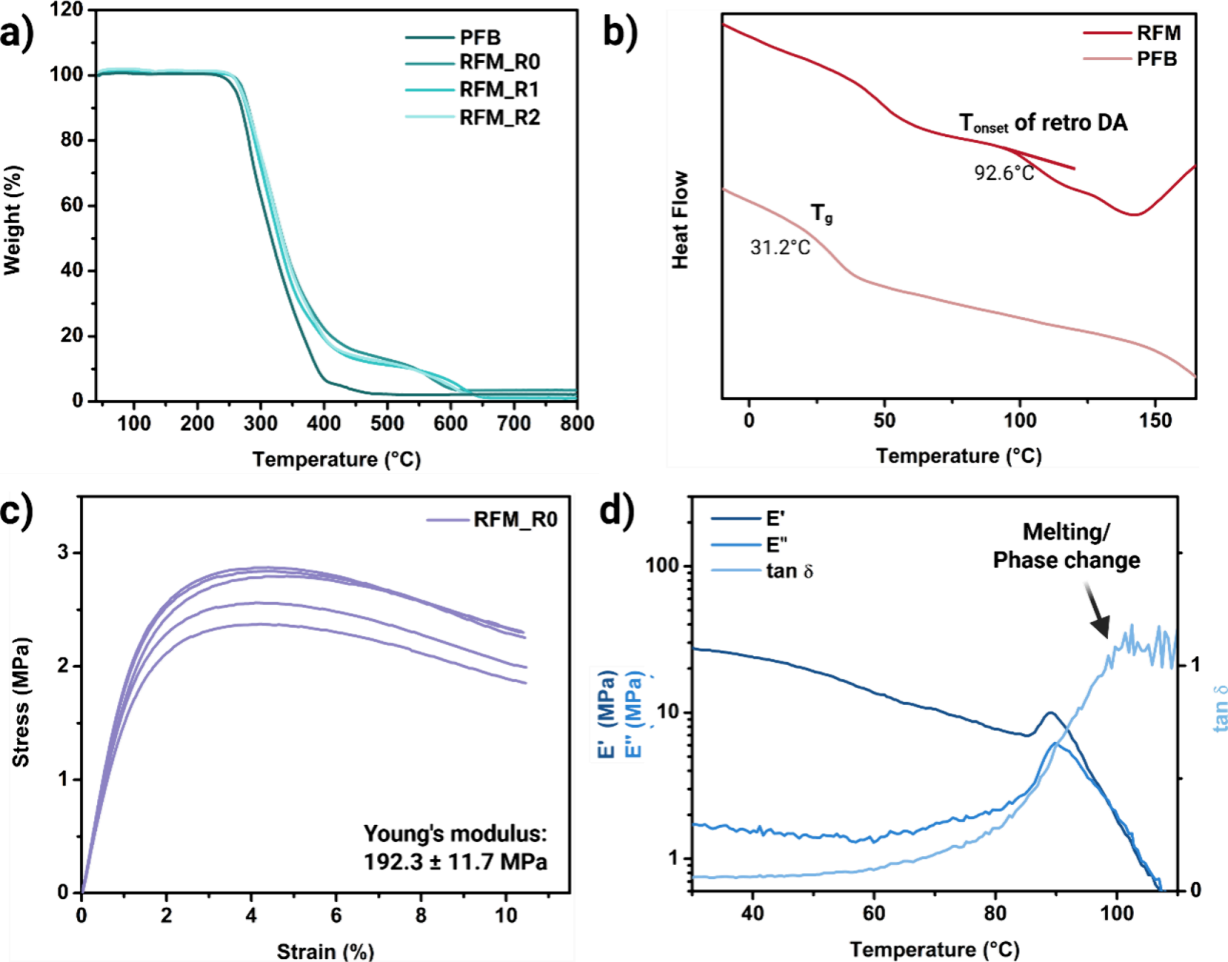
(a) TGA graph of PFB and RFM at different recycling times under atmospheric conditions. (b) DSC graph for PFB and RFM_R0. (c) Stress–strain diagram and (d) temperature sweep test of RFM_R0 from DMA.

The thermal characteristics of the copolymer PFB and the fabricated membrane RFM are illustrated in Figure 3, which consists of TGA, DSC, and temperature sweep tests of the copolymer and the membrane sample.

The success of cross-linking between PFB and BMI, and the resulting polymer structure of the cross-linked RFM, was further investigated by using thermogravimetric analysis (TGA) (Figure 3a). PFB exhibited a rapid weight loss commencing around 240 °C. In contrast, RFMs initiated weight loss at a slightly elevated temperature of approximately 260 °C. This enhanced thermal stability can be attributed to the introduction of BMI cross-linking, which restricts molecular motion within the polymer network. Furthermore, RFMs demonstrated superior thermal stability in the 400–600 °C temperature range, characterized by a gradual weight loss. This behavior is likely due to the slower degradation of the cross-linked groups. The onset of this slower degradation phase, at approximately 17% of the total weight, aligns well with the estimated weight contribution of the furan group in the copolymer. Based on peak integration in Figure 1c (peak 1:peak3), the molar ratio of furan to butyl groups was determined to be approximately 1:5.73, corresponding to a furan group weight percentage of approximately 15%.

Figure 3b presents the differential scanning calorimetry (DSC) curves for both the PFB copolymer and the RFM membrane samples. Evidence of successful cross-linking is demonstrated by the distinct *T*_*g*_: while the uncross-linked PFB copolymer exhibits a *T*_*g*_ of 31.2 °C, the *T*_*g*_ for cross-linked RFM membranes rises significantly, ranging between 90 and 140 °C. However, the steep transition observed in the DSC curve within this range does not directly indicate a precise *T*_*g*_ for the cross-linked material,^28^ as it likely reflects the onset of a retro-Diels–Alder (retro-DA) reaction, starting around 92.6 °C. Further experiments were conducted to precisely determine the *T*_*g*_ of the cross-linked membranes.

The mechanical integrity and *T*_*g*_ of a polymeric material can be determined by the dynamic mechanical analysis (DMA) technique. From the stress–strain diagram, it can be observed that the resulting RFM_R0 membranes exert high mechanical strength with a Young’s modulus of 192.3 ± 11.7 MPa, tensile strength of 2.68 ± 0.19 MPa, and elongation at break of 10.4 ± 0.1% (Figure 3c). This excellent mechanical property of the electrospun membranes is attributed to the complete DA reaction and the covalent cross-linking nature between the nanofibers. The DMA data show a peak in the loss modulus (*E”*) around 90 °C and in tan δ around 100 °C, indicating maximum energy dissipation, which closely corresponds to *T*_*g*_. Some studies also use the fluctuations of the tan δ peak as an indicator for the temperature of the retro-DA reaction, where the damping behavior becomes erratic as the structure loses its integrity by material softening or melting.^45^ Thus, the *T*_*g*_ of the RFM_R0 material is estimated to lie within the range 90–100 °C, which is also likely the onset temperature range for the retro-DA reaction. A gradual decline in storage modulus (*E’*) is observed from room temperature, a typical behavior in semiamorphous polymers like PMMA.^46,47^ This reduction in stiffness results from the increased segmental motion of the polymer chains, which intensifies as the material approaches and enters the glass transition region.

### RFM Membrane Exhibits Excellent Performance in Oil–Water Separation

To assess the performance of the as-fabricated RFM membranes, oil–water separation experiments were conducted using gravity-driven separation. Initial testing was performed with the RFM_R0 membrane using pure water (dyed with methylene blue) to demonstrate that the membrane was impermeable to the water phase (Figure 4a, Video S1). After 3 min, a heavy oil, dichloromethane (DCM, dyed with oil red O), was added to the system, forming a biphasic mixture in which water and DCM settled as the upper and lower layers, respectively, due to their density differences and immiscibility (Videos S3 an S4). In contrast to the water phase, the oil phase readily permeated the hydrophobic RFM_R0 membrane as soon as it settled beneath the water phase. Further tests assessed the pure oil flux of RFM_R0 by passing three oils—hexane, petroleum ether, and DCM—through the membrane under gravity filtration. The membrane demonstrated excellent oil flux rates of 474 LMH, 641 LMH, and 1187 LMH for hexane, petroleum ether, and DCM, respectively (Figure 4b, Video S2).

**Figure 4:**
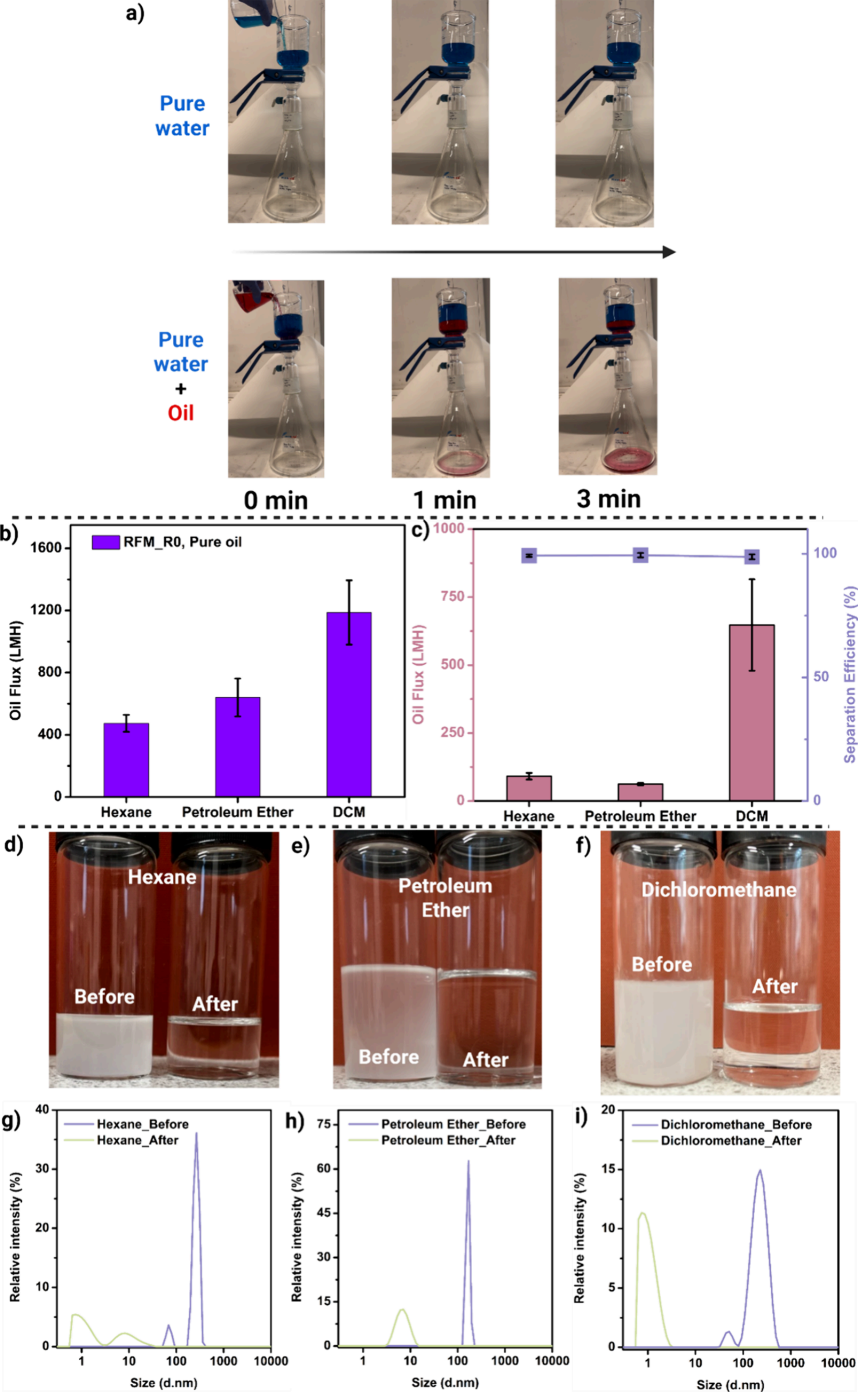
(a) Captured image of the oil–water separation test using RFM_R0. (b) Pure oil permeation test and (c) water-in-oil emulsion separation performance of the RFM_R0 membrane. Captured image of the water-in-oil emulsion separation tests using (d) hexane, (e) petroleum ether, and (f) DCM for the RFM_R0 membrane. The particle size distribution of the emulsion droplets before and after the RFM_R0 emulsion separation for (g) hexane, (h) petroleum ether, and (i) DCM systems.

The significantly enhanced permeation performance of the membrane for DCM compared to that of hexane and petroleum ether can be attributed to multiple factors. The primary driving force behind the high flux of DCM is its higher density (1.33 g cm^–3^) relative to hexane (0.60 g cm^–3^) and petroleum ether (0.60–0.70 g cm^–3^), which contributes to a greater pressure-driven transport through the membrane. Additionally, the low viscosity of DCM (0.41 mPa·s) facilitates increased permeation, as the viscosity directly influences solvent flux. Beyond viscosity, differences in the molecular size and solvent-membrane interactions play a crucial role in the observed flux variation. Despite its slightly higher molecular weight compared to hexane and petroleum ether, DCM’s compact molecular structure enhances its diffusion through the membrane. Furthermore, DCM’s greater polarity leads to stronger interactions with the membrane material, further promoting transport. These combined factors contribute to the superior permeation performance of DCM in comparison with the other solvents evaluated.

The oil–water emulsion separation performance of the RFM_R0 membrane is illustrated in Figure 4(c–i) (Video S5). Using a gravity-driven method similar to that used for pure oil flux testing, the emulsion separation capabilities of the membrane were evaluated with emulsified water-in-oil systems prepared with hexane (Figure 4d), petroleum ether (Figure 4e), and DCM (Figure 4f). By using the Karl Fisher titration method, results demonstrate that the nanofibrous RFM-R0 membrane achieves a high flux of 647 LMH and an impressive separation efficiency of approximately 98.8% for DCM emulsions (Figure 4c). In contrast, emulsified hexane and petroleum ether systems yielded significantly lower fluxes, at 91 and 62 LMH, respectively, compared to DCM. This huge discrepancy is attributed to DCM’s higher density, which reduces fouling by preventing a blocking layer from forming on the membrane surface. Despite the lower fluxes observed with hexane and petroleum ether, the membrane maintained high separation efficiencies, achieving 99.2 and 99.5%, respectively, for these emulsions.

Simultaneously, the particle size distribution of the water droplets in the emulsion both before and after was further analyzed via dynamic light scattering (DLS). The water-in-oil emulsion feeds contain water droplets with a mean particle size of around 200–300 nm, whereas all the permeates displayed a significantly narrowed droplet size distribution and lower mean particle size at around 1–10 nm (Figure 4g–i). The initial opaque emulsions were filtered into exceptionally clear and transparent filtrates, highlighting the efficacy of the membrane separation process. These findings confirm the effectiveness of the recyclable nanofibrous RFM-R0 membrane in oil–water separation applications, underscoring its potential utility across diverse fields and industrial applications.

The unusual reduction in flux for water-in-oil emulsions of hexane and petroleum ether, compared to their pure solvent systems, is primarily due to increased viscosity, membrane pore blocking, and stabilized emulsions. The presence of dispersed water droplets increases the overall viscosity, introducing greater flow resistance and impeding solvent transport. The stability of these emulsions further increases the flux reduction by maintaining water droplet dispersion and preventing phase separation. This can be observed in the sharper peaks of both hexane and petroleum ether emulsion systems in the particle size distribution figures (Figure 4g–i). In contrast, DCM, with its higher density, lower viscosity, and better membrane affinity, exhibits a smaller flux decline under similar emulsified conditions.

The structural stability and durability of the polymeric network of the nanofibrous membrane were evaluated through SEM before and after oil–water emulsion separation (Figure S4). The membrane samples maintained their fibrous architecture after exposure to the oil–water emulsion, with no evidence of catastrophic fiber breakage, tearing, and collapse. Although minor densification was observed, likely due to polymer softening or thermal shrinkage from thermally drying the membrane during sample preparation, the overall network integrity and fiber continuity were preserved. The absence of severe structural deformation suggests the cross-linked network provides sufficient mechanical strength and structural stability under gravity-driven filtration conditions.

### RFM Demonstrates Superior Closed-Loop Recyclability

To address the environmental concerns associated with the disposal of membranes, the development of closed-loop recyclable membranes is critically important. To simulate realistic conditions, we intentionally contaminated the membrane samples with oil and sand (Figure 5a). Subsequently, the contaminated membranes were heated in DMF at 140 °C to facilitate depolymerization. Large particulate contaminants were removed by filtering the resulting dope solution through filter paper, while the immiscibility of oil with DMF enabled its efficient separation. The processed dope solution was then repurposed to fabricate a new recyclable membrane via electrospinning, using the same parameters as those employed for the pristine RFM_R0.

**Figure 5:**
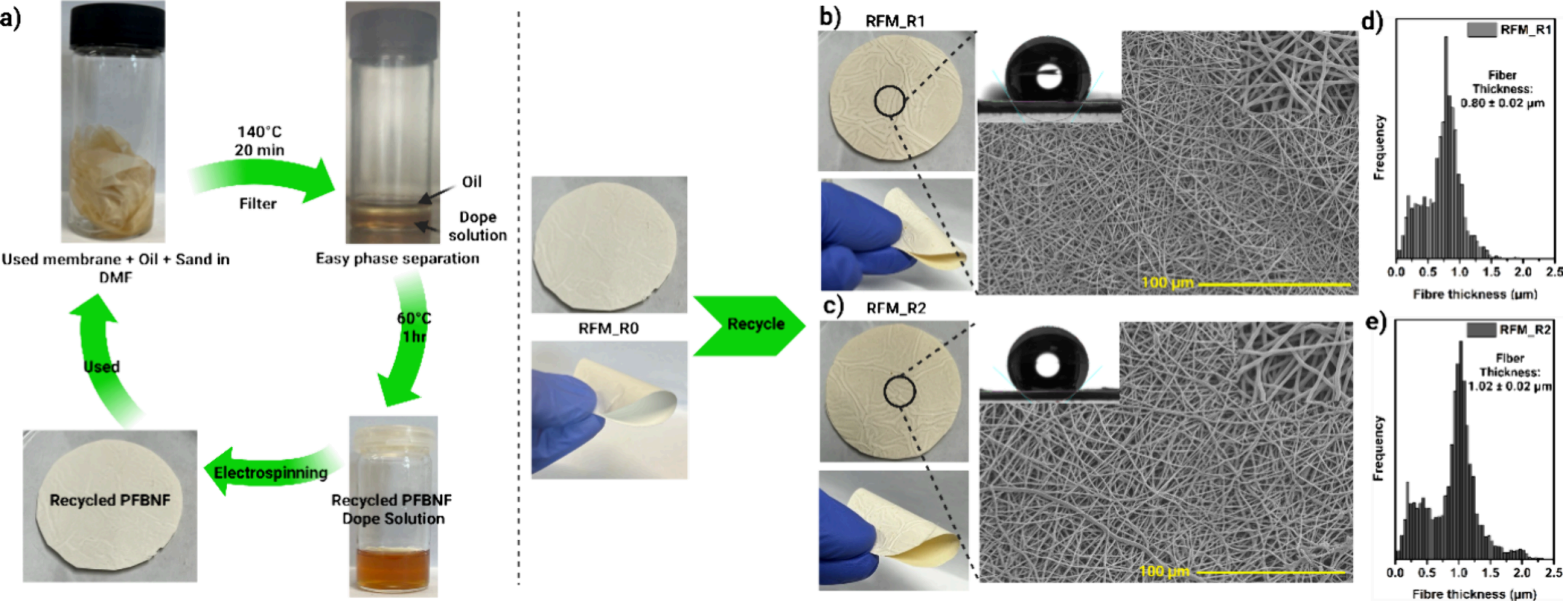
(a) Schematic diagram of the recycling step for the RFM membranes. Captured images SEM images of (b) RFM_R1 and (c) RFM_R2. Distribution of the fiber diameters for (d) RFM_R1 and (e) RFM_R2.

The pristine RFM_R0 exhibits an off-white appearance (Figure 2a), while the recycled membranes, RFM_R1 and RFM_R2, display a yellowish off-white color compared to the pristine membrane (Figure 5b,c). This slight discolouration is likely attributed to the oxidation of the maleimide and furan groups and the self-reaction of the maleimide group during the retro-DA reaction that occurs under high-temperature conditions during recycling. The oxidation phenomena can be confirmed via XPS with the increase in the atomic percentage of the O 1s after recycling, where the O concentration increases from 17.68 to 19.80% after the first recycling process and is later maintained at around 18.64% after the second recycling process (Figure 6c). The FT-IR spectra in Figure 6a did not show any obvious emergence or diminishing peaks in the recycled samples, which can be due to the low concentration of oxidizing groups or overlapping with the existing peaks, such as the carbonyl groups at ∼1730 cm^–1^. Moreover, the self-reaction of the maleimide group from BMI can occur at temperatures close to 150 °C, which can cause an irreversible reaction of BMI, leading to lower retro-DA reactions.^48^

**Figure 6:**
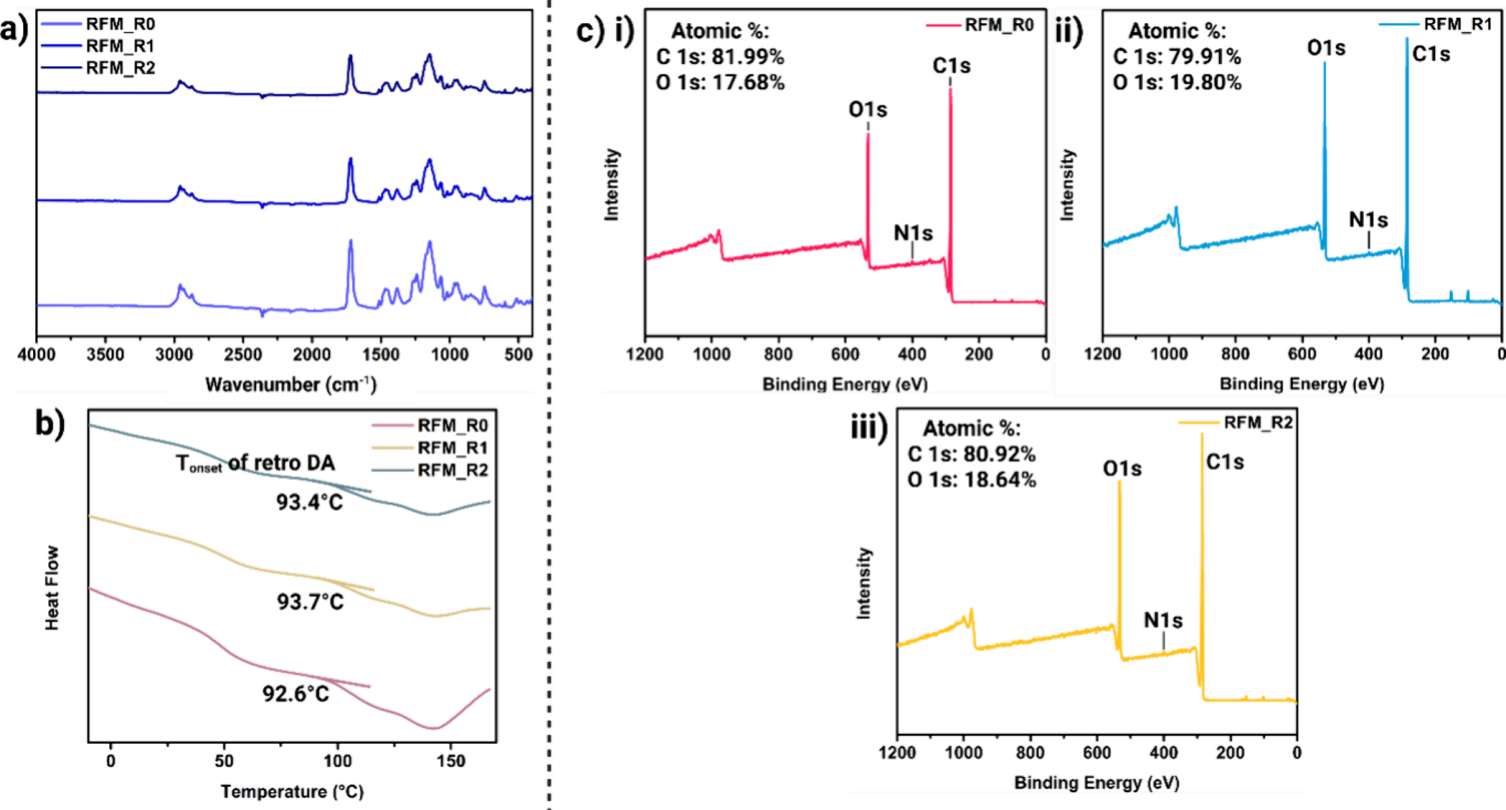
(a) FT-IR spectra and (b) DSC graph of RFMs. (c) Wide XPS spectra of (i) RFM_0, (ii) RFM_R1, and (iii) RFM_R2.

During the recycling process, the dope solution concentration was adjusted to 28 wt %, which was slightly lower than the original weight concentration. Consequently, the average fiber diameter of RFM_R1 was reduced to 0.80 μm compared to 1.05 μm for the pristine RFM_R0 (Figure 5d and Table 1), which led to a slight reduction in the water contact angle. However, following a second recycling cycle, the average fiber diameter increased to 1.02 μm (Figure 5e), resulting in a higher water contact angle, which is caused by the increased surface roughness. Importantly, the chemical environment of the RFMs remained largely unchanged after recycling, as evidenced by the FT-IR spectra (Figure 6a). Additionally, the degradation behavior (Figure 3c) and the retro-DA reaction onset temperature (Figure 6b) showed no significant changes, showing the excellent thermal stability of the RFMs even after multiple recycling cycles. Nevertheless, a reduction in mechanical integrity was observed in the recycled membranes (Table 1, Figure S4). This decline is likely due to the oxidation of maleimide and furan groups and the self-reaction of the maleimide group from BMI during the retro-DA reaction. In addition, the membrane thickness, fiber diameter and diameter distribution, fiber orientation, and polymer structure are also the main parameters that affect the mechanical integrity.^49,50^ It is also noteworthy that this decline in tensile strength was sufficient for the gravity-driven filtration performance test of the membrane, and it can still be bent easily without any breakage, as shown in Figure 5b,c. However, this will still be a challenge for this material as this will pose a significant downside when recycling is considered for the synthesis of the material and the fabrication of the membrane.

**Table 1:** Key Mechanical Properties of RFM before and after Recycling

	contact angle (°)	fiber thickness (μm)	tensile strength (MPa)	Young’s modulus (MPa)
RFM_R0	128.4 ± 2.3	1.05 ± 0.42	2.68 ± 0.19	192.3 ± 11.7
RFM_R1	126.0 ± 6.9	0.80 ± 0.02	1.44 ± 0.19	47.6 ± 4.8
RFM_R2	133.1 ± 4.6	1.02 ± 0.02	0.99 ± 0.15	37.3 ± 5.4

The performance of the recycled membranes was systematically evaluated. The pure oil flux of the RFMs exhibited a slight increase after the first recycling cycle but declined following the second cycle (Figure 7a), with a similar trend observed during the water–oil emulsion separation (Figure 7b). These variations between the pristine and recycled membranes are likely due to changes in fiber diameter, porosity, and membrane thickness introduced during the recycling process. Notably, despite these flux fluctuations, the water–oil emulsion removal efficiency remained consistent across recycling cycles, demonstrating excellent separation performance even after repeated use. This consistency in structure and performance highlights the potential of RFMs as sustainable, closed-loop alternatives for oil–water separation.

**Figure 7:**
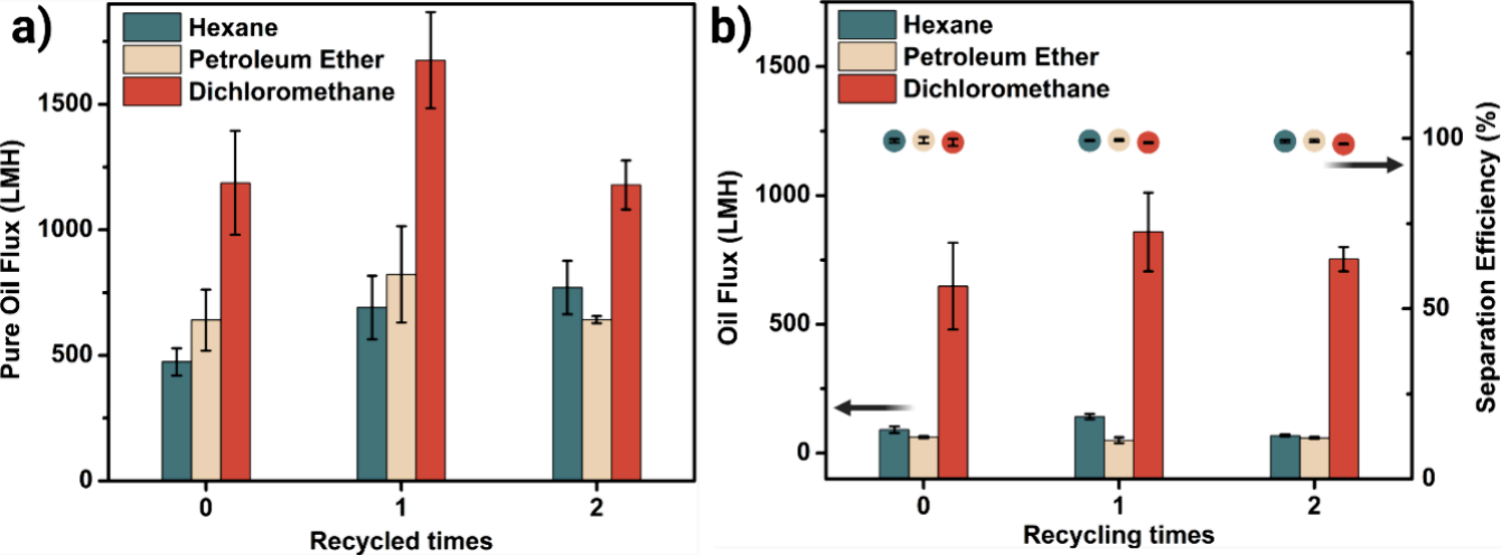
(a) Pure oil flux and (b) water emulsion separation performance of RFMs.

To further evaluate the recyclability of the RFMs, the membranes were recycled a third time. For the electrospinning of RFM_R3, the dope solution concentration was adjusted to 26 wt %. After fabrication, the recycled nanofibrous membrane (RFM_R3) exhibited a more pronounced yellowish color compared to previous generations, possibly indicating increased oxidation within the polymer structure (Figure S6a). Additionally, the fibers in RFM_R3 appeared less uniform and significantly thinner, likely due to the reduced dope concentration and structural inconsistencies within the cross-linked polymer network (Figure S6b). he water contact angle of RFM_R3 was measured at 123.6 ± 0.8°, showing a decreasing trend compared to RFM_R2. This reduction is potentially attributed to the decreased surface roughness resulting from the smaller fiber diameters.

Notably, FT-IR spectroscopy revealed no significant changes in the functional groups of the polymer across RFM_R0, RFM_R1, RFM_R2, and RFM_R3, further supporting the high recyclability of the RFMs (Figure S6c). The filtration performance of RFM_R3 was evaluated by using pure DCM and DCM-water emulsions and compared to that of previous recycled forms. The pure oil flux of RFM_R3 increased from 1178 LMH (RFM_R2) to 1574 LMH (Figure S6d), attributed to the increased membrane porosity resulting from the thinner fiber structure.^51^ Similarly, in emulsion separation, the flux increased from 753 to 824 LMH (Figure S6e), indicating improved throughput. However, the separation efficiency decreased from 98.9 to 97.0%, likely due to the increased porosity and reduced water contact angle. Overall, these results demonstrate that the RFMs maintain excellent recyclability, preserving their structural integrity and functional performance even after multiple recycling cycles.

### Environmental Implications

These findings underscore the potential of RFMs as a sustainable, closed-loop solution for oil–water separation, combining high separation efficiency with recyclability. By significantly reducing the waste generated by end-of-life membranes, this approach can help lower the carbon footprint associated with membrane disposal. Moreover, the versatility of RFMs opens opportunities for application in other wastewater treatment systems, such as the removal of micropollutants and dyes. To further enhance the membrane’s efficiency for removing aqueous contaminants, incorporating monomers with hydrophilic groups into the copolymer synthesis could be explored. By introducing hydrophilic groups into the molecular structure, water-based contaminants like dyes will be more likely to latch onto the polymer, and the aqueous phase could easily pass through the membrane, thereby increasing the removal rate and flux of the membrane simultaneously.

## Conclusions

Results reported here highlight the successful development of a high-performance, closed-loop recyclable nanofibrous membrane capable of efficiently separating oil–water emulsions, even after undergoing recycling. The recycling process leverages the dynamic nature of the DA reaction, enabling the depolymerization of covalent bonds at high temperatures and their subsequent reformation at lower temperatures. A methacrylate random copolymer containing butyl and furfuryl groups was synthesized via free radical polymerization and cross-linked with bismaleimide to form a DA adduct. This polymer was then fabricated into RFMs through electrospinning, resulting in membranes with excellent mechanical integrity and thermal stability. The pristine membrane (RFM_R0) demonstrated outstanding performance in pure oil separation (474–1187 LMH) and oil–water emulsion systems (flux: 62–647 LMH, water removal rate: 98.8–99.5%). After depolymerization of the dope solution at elevated temperatures, the membranes were refabricated into RFM_R1 and RFM_R2. Remarkably, the recycled membranes retained most of the structural and chemical properties of the pristine membrane and exhibited a comparable performance in terms of flux and water removal efficiency.

Despite these successes, the decline in the mechanical integrity of the RFMs after recycling poses a challenge that needs to be addressed, particularly for potential scale-up applications. Additionally, the polydispersity index of the copolymer could be reduced by optimizing the polymer synthesis parameters to achieve a more uniform and consistent polymer structure. This improvement in the polydispersity index could potentially lead to a polymer with more control over its molecular weight, thereby increasing the simplicity of manipulating the mechanical strength of the polymer.

## Supplementary Material













## Notes

1H NMR graph for PFB copolymer with integration of furfuryl and butyl groups, gel content test of RFM_R0, XPS spectra of the RFMs at their relevant core levels (C, N, O), SEM image of RFM_R0 before and after hexane-water and DCM-water emulsion, stress–strain diagrams for different RFMs, and captured image, SEM, FTIR spectra, filtration, and filtration performance for RFM_R3 (PDF)
Permeation performance of RFM_R0 on pure water (dyed blue) (MP4)
Permeation performance of RFM_R0 on DCM (dyed red) (MP4)
Permeation performance of RFM_R0 on DCM (dyed red)/water (dyed blue) (before) (MP4)
Permeation performance of RFM_R0 on DCM (dyed red)/water (dyed blue) (after) (MP4)
Permeation performance of RFM_R0 on the DCM/water emulsion (MP4)
