# Supplementary material for: Dynamic Covalent Chemistry Enabled Closed-Loop Recycling of Thermally Modified Polymer Membrane
Source: ACS Appl Polym Mater. 2025 Jun 17;7(12):7824–35. doi: 10.1021/acsapm.5c00491 (PMC12210218; doi:10.1021/acsapm.5c00491)
Supplement: Supplementary file 1 [file ap5c00491_si_001.pdf]

Supporting Information

Dynamic Covalent Chemistry Enabled Closed-loop Recycling of Thermally Modified Polymer Membrane

Submitted to

ACS Applied Polymer Materials

Ching Yoong Loh[a], Tianting Pang[a,b], Dengsong Zhang[b], Andrew D. Burrows[c] , Ming Xie[a*]

a Department of Chemical Engineering, University of Bath, Bath, BA2 7AY, United Kingdom

b Department of Chemistry, College of Sciences, Shanghai University, Shanghai, 200444, PR China

c Department of Chemistry, University of Bath, Bath, BA2 7AY, United Kingdom

*Corresponding author: E-mail: m.xie2@bath.ac.uk (M. Xie)

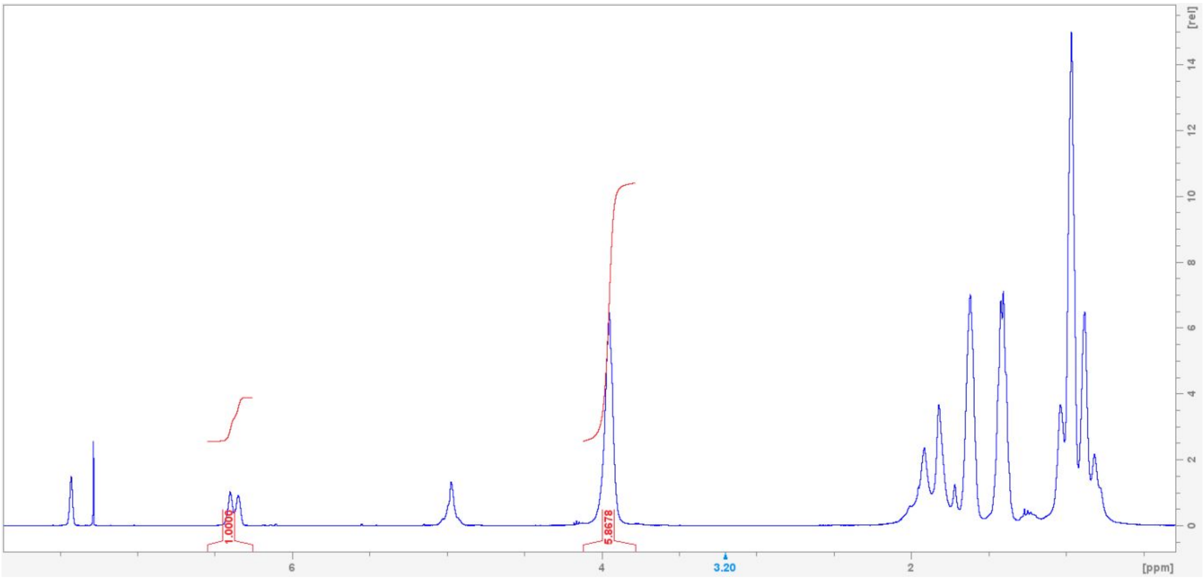

Figure S1 ^{1}H NMR graph for PFB polymer with integration of furfuryl and butyl groups.

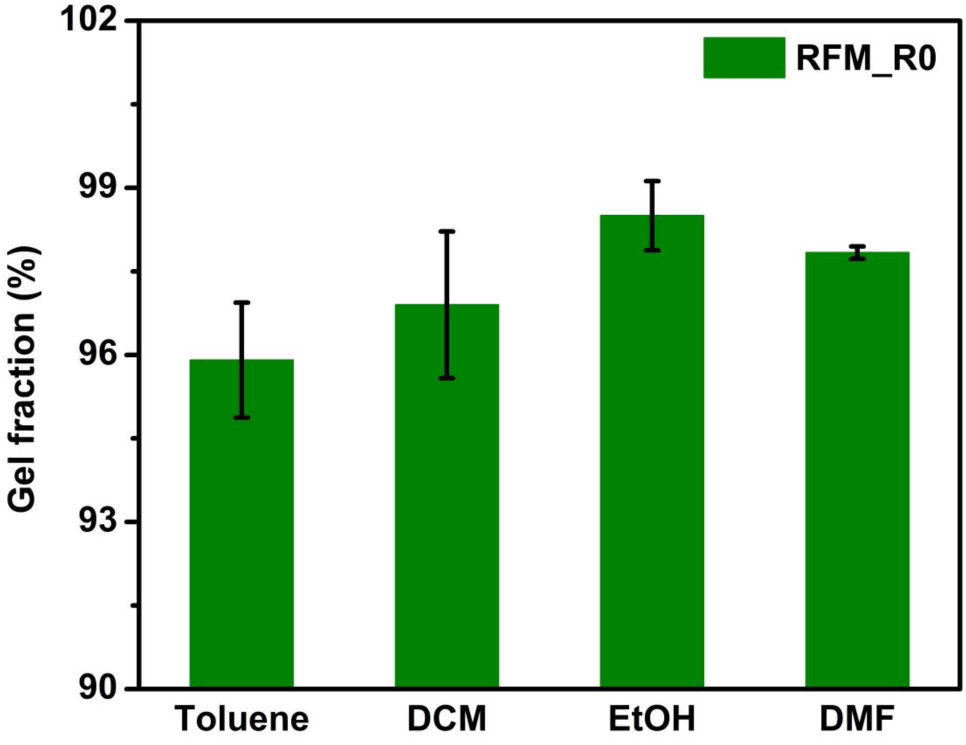

Figure S2 Gel content test of RFM membrane with different organic solvents.

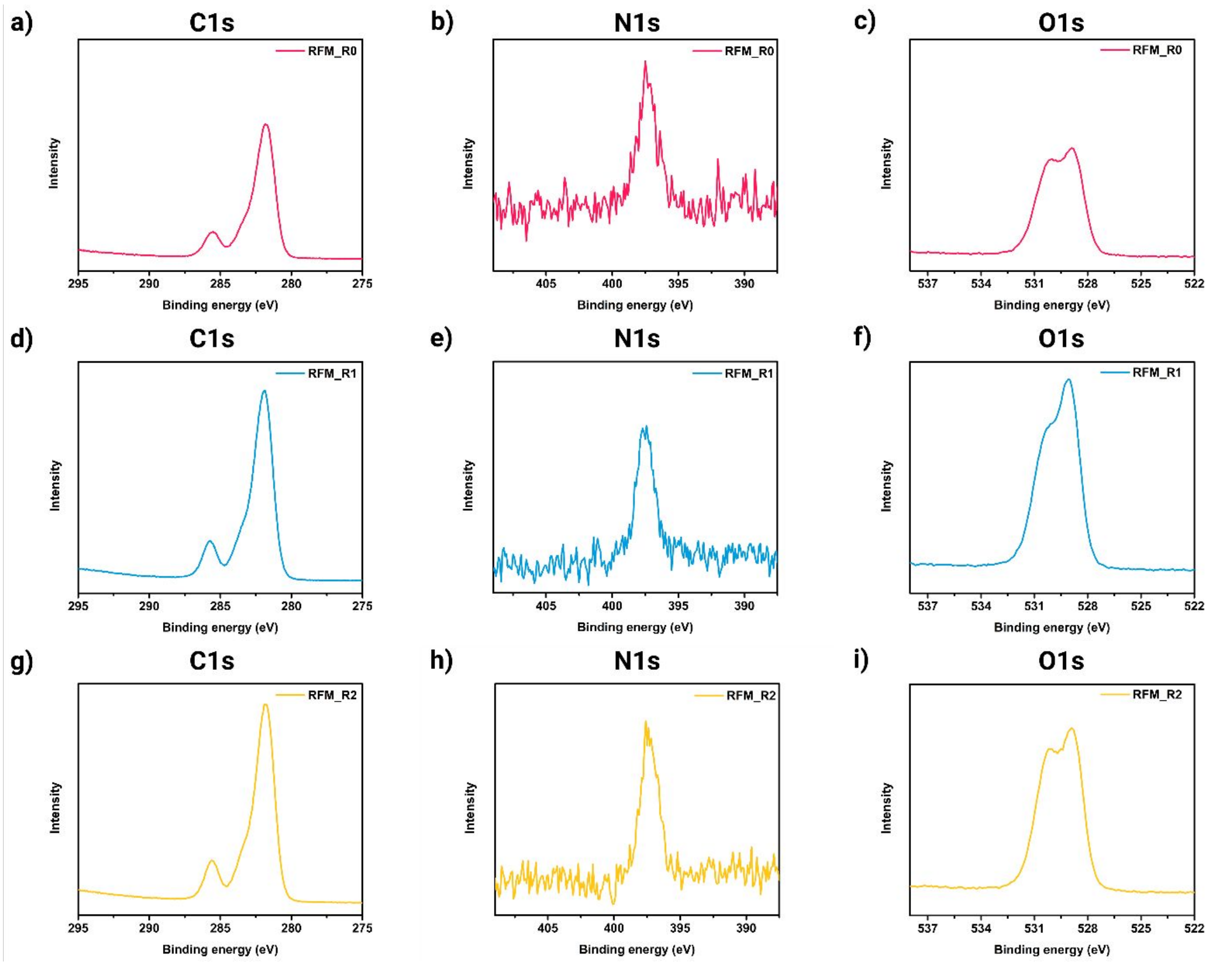

Figure S3 (a) C 1s, (b) N 1s and (c) O 1s XPS spectra of RFM_R0. (d) C 1s, (e) N 1s and (f) O 1s XPS spectra of RFM_R1. (g) C 1s, (h) N 1s and (i) O 1s XPS spectra of RFM_R2.

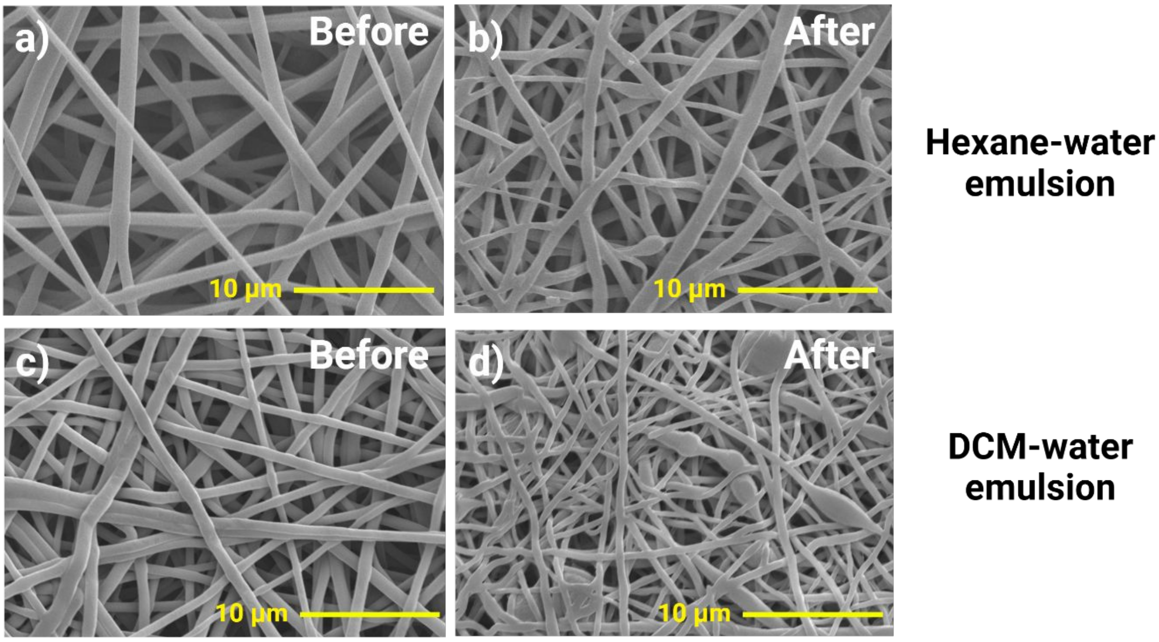

Figure S4 SEM images of RFM_R0 before (a, c) and after (b, d) hexane-water emulsion and DCM-water emulsion filtration.

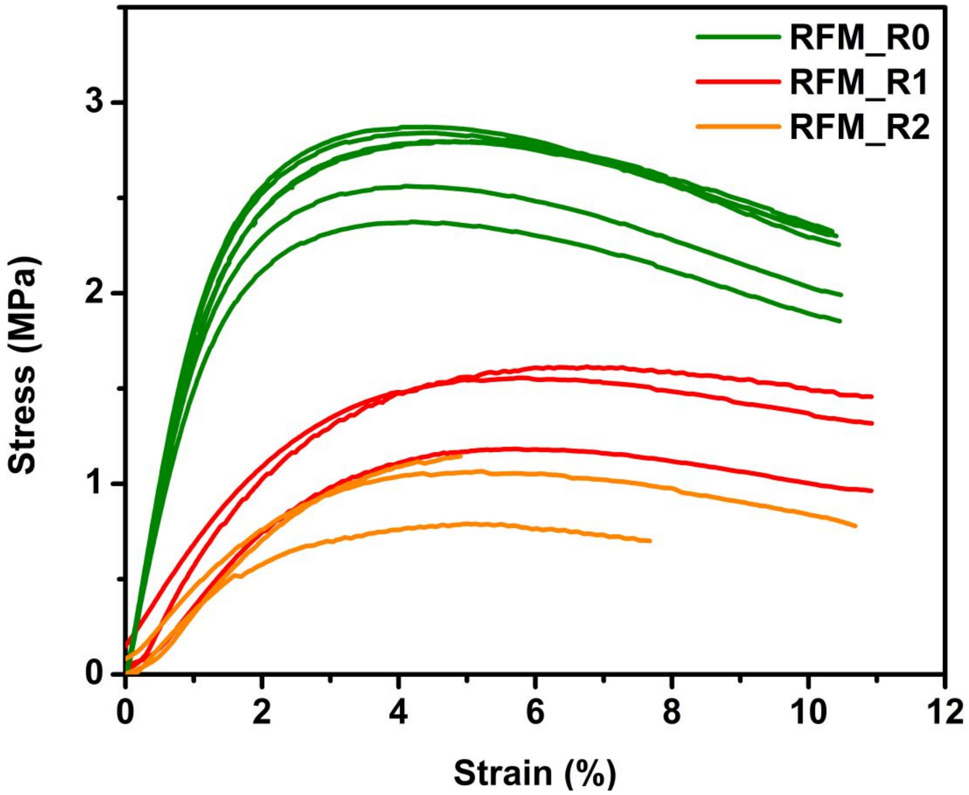

Figure S5 Stress-strain diagram of the RFMs at different recycling times.

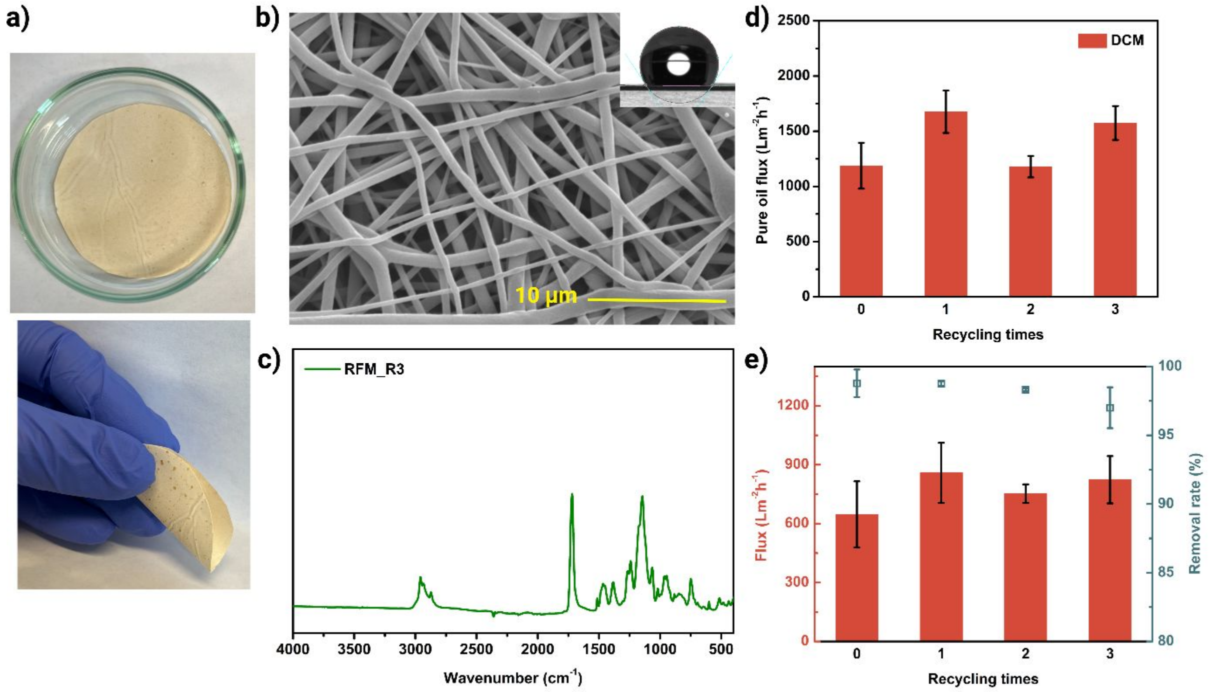

Figure S6 (a) The captured image, (b) SEM image and (c) FTIR spectra of RFM_R3. (d) The filtration performance of the recycled membrane using (d) DCM for pure oil tests and (e) DCM-water emulsion system for emulsion separation tests.

Video S1 The permeation performance of RFM_R0 on pure water (dyed blue).

Video S2 The permeation performance of RFM_R0 on DCM (dyed red).

Video S3 The permeation performance of RFM_R0 on DCM (dyed red)/water (dyed blue) (before).

Video S4 The permeation performance of RFM_R0 on DCM (dyed red)/water (dyed blue) (after).

Video S5 The permeation performance of RFM_R0 on DCM/water emulsion.
